# Effects of curcumin on global gene expression profiles in the highly invasive human breast carcinoma cell line MDA-MB 231: A gene network-based microarray analysis

**DOI:** 10.3892/etm.2012.754

**Published:** 2012-10-19

**Authors:** NACI CINE, PORNNGARM LIMTRAKUL, DENIZ SUNNETCI, BALINT NAGY, HAKAN SAVLI

**Affiliations:** 1 Department of Medical Genetics and Clinical Research Unit, Faculty of Medicine, Kocaeli University, Kocaeli, Turkey;; 2 Biochemistry Department, Chiang Mai University, Thailand;; 3 First Department of Obstetrics and Gynecology, Genetics Laboratory, Semmelweis University, Budapest, Hungary

**Keywords:** curcumin, microarray, gene expression, breast cancer

## Abstract

Curcumin, or diferuloylmethane, is a major chemical component of turmeric (*Curcuma longa* Linn.) that has been consumed as a dietary spice through the ages. This yellow-colored polyphenol has a notably wide range of beneficial properties, including anti-inflammatory, antioxidant, antitumoral, anti-invasive and anti-metastatic activity. In the present study, microarray gene expression analysis was applied to identify the curcumin-regulated genes in a highly invasive human breast carcinoma cell line (MDA-MB 231). Cells were cultured with curcumin (20 *μ*M) for 24 h; total RNA was isolated and hybridized to Whole Human Genome Microarray slides. Gene set enrichment analyses on our whole genome expression data revealed downregulation of the EGF pathway elements following curcumin treatment. Furthermore, gene network analysis identified a significantly relevant network among the differentially expressed genes, centered on the *EGR1* and *FOS* genes. The members of these pathways and networks play an essential role in the regulation of cancer cell growth and development; the majority exhibited decreased expression levels following treatment with curcumin. These observations suggest that curcumin is an excellent candidate for the prevention and treatment of breast cancer.

## Introduction

The polyphenol curcumin is the active ingredient in the herbal remedy and dietary spice turmeric (*Curcuma longa* Linn.). Turmeric has been widely used in traditional Indian medicine to cure biliary disorders, anorexia, coughs, diabetic wounds, hepatic disorders, rheumatism and sinusitis since the time of Ayurveda (1). Extensive research over the last decade has revealed that this molecule is capable of reducing blood cholesterol (2), preventing low-density lipoprotein oxidation (3) and inhibiting platelet aggregation (1). Curcumin is capable of exerting a wide range of antiproliferative and proapoptotic effects against various tumors *in vitro*(4–6) and *in vivo*. The compound has been identified as being able to successfully prevent carcinogenesis of the breast (7) and various other organs (8–10), targeting multiple steps in the pathway to malignancy. Additionally, curcumin has been shown to suppress thrombosis (11) and myocardial infarction (12) and to suppress symptoms associated with type II diabetes (13) and rheumatoid arthritis (14). These pleiotropic molecular effects may be mediated by the inhibitory effect curcumin has on a wide variety of cell signaling factors, including AP-1 transcription factor, c-Myc, EGR-1, NF-κB, protein kinase C, epidermal growth factor receptor tyrosine kinase, c-Jun N-terminal kinase, protein tyrosine kinases, protein serine/threonine kinases and IκB kinase (15–17).

Metastasis is a complex integrated process that involves interactions between cancer cells and their surrounding microenvironments, and it is one of the most significant deciding factors in the outcome of cancer. The majority of drugs currently available for the treatment of cancer have limited potential as they are extremely toxic, highly inefficient in treating cancer or highly expensive. Therefore, treatments without these disadvantages are certainly required. Curcumin is one such agent; several studies have shown that curcumin is able to kill cancer cells selectively (18–20). Curcumin has previously been identified to exhibit antimetastatic activity in *in vivo* animal models (21,22). Oral administration of curcumin was revealed to significantly inhibit lung metastasis (21), and curcumin treatment significantly inhibited the invasion of B16F-10 melanoma cells (22). These results indicate a possible use of curcumin as an antimetastatic agent, but the mechanism involved behind these beneficial effects of the ideal ‘Spice for Life’ remains unknown.

The advantages of high-throughput microarray technologies offer a new opportunity to gain insight into the global gene expression changes induced by curcumin in various highly metastatic carcinoma cell lines, leading to the identification of new curcumin-regulated genes and pathways. Previously, researchers observed an anti-invasive gene expression profile following curcumin treatment in lung adenocarcinoma, based on a cDNA microarray analysis (23). These studies also highlighted that several additional, and as of yet unidentified, gene interactions may be responsible for the multiple beneficial effects of curcumin. The aim of our current study was to identify novel curcumin-regulated genes, gene networks and pathways in a highly invasive human breast carcinoma cell line (MDA-MB 231) by applying microarray gene expression analysis subsequent to 24 h of curcumin treatment. Furthermore, to enable the integration of our results into multiple levels of information available in public databases, we applied gene set enrichment analysis (GSEA) and gene network analysis to our microarray data.

## Materials and methods

### Materials

Dulbecco’s modified Eagle’s medium (DMEM), penicillin-streptomycin and trypsin-EDTA were purchased from Gibco-BRL (Grand Island, NY, USA). Fetal calf serum (FCS) was purchased from Hyclone (Logan, UT, USA). MTT (3-(4,5-dimethylthiazol-2-yl)-2,5-diphenyltetrazolium bromide) was purchased from Sigma (St. Louis, MO, USA).

### Cell line and culture conditions

The MDA-MB 231 human invasive breast carcinoma cell line was purchased from the American Type Culture Collection (ATCC; Manassas, VA, USA). The cells were grown in Leibovitz L-15 medium supplemented with 100 U/ml penicillin, 100 mg/ml streptomycin and 10% heat-inactivated FCS. The cultures were maintained at 37°C in a humidified atmosphere without CO_2_.

### Extraction and isolation of curcuminoids

Curcumin was isolated and purified as previously described (24). Briefly, Chiang Mai turmeric powder (1 kg) was successively extracted with hexane (2.5 l) and 95% ethanol (7.5 l) at room temperature. Turmeric curcuminoids were then precipitated with petrolium ether yielding 50 g crude curcuminoid mixtures (78% curcumin, 16% demethoxycurcumin and 5% bisdemethoxycurcumin). The curcuminoids (3 g) were further fractionated by Silica gel 60 column chromatography (44×1.6 cm) using first CHCl_3_ and then CHCl3/MeOH with increasing polarity. Fractions containing curcumin (1.11 g) were eluted with 100% CHCl_3_ (0.6 l). Fractions containing demethoxycurcumin (200 mg) and bisdemethoxycurcumin (40 mg) were further eluted with CHCl_3_/MeOH (98:2, 0.8 l) and CHCl_3_/MeOH (95:5, 1 l), respectively.

### MTT assay of cell viability

Cell viability was measured using the conventional MTT reduction assay as described previously (25). Briefly, MDA-MB 231 cells were inoculated at a density of 5×10^3^ cells/well in 96-well plates for 24 h, in 200 *μ*l DMEM with 10% FCS. The culture supernatant was removed and DMEM with 10% FCS containing various concentrations of curcumin was added and incubated for 24 h. MTT dye (10 *μ*l, 5 mg/ml) was added and the plate was incubated for an additional 4 h. The absorbance of MTT-formazan was measured using a microplate reader at 540 nm with a reference wavelength of 630 nm.

### Preparation of cell pellets

MDA-MB 231 cells were seeded into a 75 mm^3^ T flask in DMEM with 10% FCS. Subconfluent cell cultures were incubated for 24 h in nontoxic concentrations of curcumin (20 *μ*M) in DMEM with 10% FCS. Following treatment, the cells were washed twice with ice-cold PBS and then scraped with a cell scraper into further ice-cold PBS. The cells were centrifuged at 500 × g for 10 min, the supernatant was removed and the cell pellets were lysed.

### RNA isolation, quality and quantity determination

Cellular total RNA was prepared by RNeasy columns (Qiagen, Valencia, CA, USA). The quality and quantity of total RNA was determined with an Agilent 2100 Bioanalyzer (Agilent Technologies, Palo Alto, CA, USA). Only those samples that yielded >8.0 for RNA integrity number, exhibited a clear gel image and for which no DNA contamination was observed on the histogram, were used for microarray or real-time PCR experiments.

### Quantitative real-time PCR (Q-RT-PCR)

cDNA was synthesized using DNase-I-treated total RNA with a First Strand cDNA Synthesis kit for RT-PCR (Roche Diagnostic Corp., Indianapolis, IN, USA) according to the manufacturer’s instructions. Q-PCR for determination of *FOS*, *EGR1* and GDF15 gene expression was performed using a LightCycler (Roche Diagnostic Corp.) as described previously (26). The G6PD gene was used as an internal control. The gene sequences are shown in Table I.

### Microarray measurements, data normalization and analysis

The 1000 ng quality-checked total cellular RNA was reverse transcribed using a Low RNA Input Linear Amplification kit (Agilent Technologies) and then transcribed to Cy3-labeled cRNA according to the manufacturer’s instructions. The labeled cRNA was purified (RNeasy kit; Qiagen) and the dye content (>9.0 pmol dye/*μ*g cRNA) and concentration of cRNA was measured using a NanoDrop ND-1000 spectrophotometer (NanoDrop Technologies, Wilmington, DE, USA). Cy3-labeled cRNA (1650 ng) was hybridized to Whole Human Genome Oligo 4×44 k microarrays overnight at 65°C, then the slides were washed and treated with Stabilizing and Drying Solution (Agilent Technologies) and scanned by an Agilent Microarray Scanner. All steps were carried out according to the manufacturer’s instructions. Data were normalized by the Feature Extraction software, version 7.5, with default parameter settings for one-color oligonucleotide microarrays and then transferred to the GeneSpring 9.02 program (Agilent Technologies) for further statistical evaluation. The normalization and data transformation steps recommended by Agilent Technologies for one-color data were applied once in GeneSpring. Experimental interpretation was built by GeneSpring, and the expressed genes that exhibited a >2.0-fold differential expression were further analyzed by statistical tests. The statistical comparisons were performed between the groups by performing an unpaired t-test. The multiple testing correction method designed by Benjamini-Hochberg was applied with a P<0.05 cut-off value in our statistical tests.

### Gene network analysis

For our proposed gene network analysis, gene symbols (Human Gene Organization; HUGO), Agilent probe IDs and the fold change of the significantly differentially regulated genes (curcumin-treated samples versus control) were imported into the IPA 5.0 software (Ingenuity Systems, Redwood City, CA, USA). In IPA, the analysis was carried out with P<0.05 as the cut-off point. Those genes with known gene symbols (HUGO) and their corresponding expression values were uploaded into the software. Each gene symbol was mapped to its corresponding gene object in the Ingenuity Pathways Knowledge Base. Networks of these genes were algorithmically generated based on their connectivity and assigned a score. The score was a numerical value used to rank networks according to their relevance to the genes in the input dataset but this may not have indicated the quality or significance of the network. The score took into account the number of focus genes in the network and the size of the network, to approximate the relevance of this network to the original list of focus genes.

### GSEA

The GSEA was carried out using GSEA v2.0 software (27). For the GSEA, a collection of canonical pathway gene sets containing 639 pathways was downloaded from the Molecular Signatures Database (MSigDB) website (http://www.broadinstitute.org/gsea/msigdb/index.jsp). The pathway gene sets were curated from the Kyoto Encyclopedia of Genes and Genomes (KEGG), BioCarta and GenMAPP online pathway databases. Usually, these gene sets are canonical representations of a biological process compiled by domain experts. Probes were collapsed to HUGO gene names. Gene sets were assessed as to whether they individually scored highly when compared with other possible choices of gene sets. This provided an unbiased means of assessing pathways and of testing gene lists with respect to the enrichment degree of representation of highly regulated genes. A positive normalized enrichment score (NES) indicated correlation with the control, while a negative value indicated correlation with curcumin treatment. The nominal P-value estimated the statistical significance of the enrichment score for a single gene set. We drew conclusions from the top gene sets that had a false discovery rate of <5% and a P-value of <0.05, both of which are acceptable cut-off values for the identification of biologically relevant gene sets.

## Results

### Measurement of cell viability

The number of viable cells following curcumin treatment for 24 h was estimated using an MTT assay. Exposure of MDA-MB 231 cells to curcumin (0, 10, 20, 30, 40 and 60 *μ*M) affected the viability of the cells in a concentration-dependent manner (96.5±4.0% at 10 *μ*M, 95.2±5.5% at 20 *μ*M, 70.3±3.1% at 30 *μ*M, 53.3±4.0% at 40 *μ*M and 24.0±0.9% at 60 *μ*M, P<0.05, ANOVA) The inhibitory concentration of cell growth at 50% was 40.0±2.8 *μ*M in the MDA-MB 231 cells (n=3).

### Microarray gene expression analysis

Following 24 h of curcumin treatment (20 *μ*M; >90% cell survival rate), 35 genes were revealed to be significantly differentially expressed relative to the control. This was tested using an Agilent Whole Human Genome (4×44 k) Microarray Platform and traditional microarray data analysis. The list of these genes is shown in Table II.

### Verification of selected genes by Q-RT-PCR

To substantiate the results of the microarray studies, Q-RT-PCR was performed to assess the mRNA expression of *FOS*, *EGR1* and *GDF15*. As presented in Table I, the microarrays and real-time PCR resulted in similar gene expression changes in the case of the three genes mentioned above, confirming the reliability of our microarray results at the mRNA level.

## Discussion

The anticancer potential of curcumin in various systems has been reviewed previously (28). Curcumin has been shown to block transformation, tumor initiation, tumor promotion, invasion, angiogenesis and metastasis. In the present study, we investigated the effect of 24-h curcumin treatment on the global gene expression profile of the MDA-MB 231 human invasive breast carcinoma cell line. Since the traditional microarray data analysis generated only a moderate number of significantly differentially regulated genes, we applied GSEA on our whole genome gene expression data; this algorithm is designed to effectively evaluate the outcome of a specific experimental condition on known biological pathways and functional categories. In numerous cases, traditional gene expression analyses of large scale microarray data focus on statistically differentially expressed genes, which may lead to a number of potentially significant disease-related genes being bypassed. GSEA is capable of solving this problem by focusing on gene sets rather than individual genes. This method tests the hypothesis of whether members of an *a priori* defined gene set (e.g., biological pathways) are enriched in the rank-ordered list. This list is generated from the whole microarray data based on the difference between two biological states (e.g., treated versus control). Our GSEA focused on predefined gene sets from pathway databases. We drew conclusions only from the top gene sets (with a false discovery rate of less than 5% and a P-value of less than 0.05, Table II). Among these, pathways associated with PDGF, EGF signaling, cell proliferation, the cell cycle and cell junctions exhibited the strongest correlation with the control group, while pathways related to hematopoietic cell lineage and B cells correlated only with the curcumin-treated group. This observation highlights the suppressive effect curcumin has on various cell proliferation pathways by decreasing the expression level of their members. In spite of this, the induction of pathways related to hematopoietic cell lineage and B cells stimulates the activation of several immune function-related cytokines and cell adhesion molecules. These immune-stimulating effects may lead to the activation and attraction of nearby immune cells surrounding the tumor tissue, enhancing the natural defense response of the body against the tumor cells.

These analyses indicate whether a given treatment (e.g., curcumin stimulation) results in enrichment of gene sets that are involved in the regulation of specific pathways (see Materials and methods for details). GSEA clearly revealed that the pathways involved in PTEN, PDGF, EGF signaling, cell proliferation and the cell cycle exhibited significant positive correlation with the control group, while none of these sets were enriched in the curcumin-treated samples, therefore indicating the suppressive effect of curcumin on various cell proliferation pathways. Gene interaction based network investigation of the 35 significantly differently expressed genes obtained by traditional microarray data analysis identified a significantly relevant gene network around *FOS* and *EGR1* genes. The members of this network play an essential role in the regulation of cancer cell growth and development; the majority exhibited decreased expression levels following treatment with curcumin.

Overexpression and aberrant function of EGFR have been identified in a variety of human tumors, including colorectal cancer (29). Activation of EGFR is initiated by binding of ligands, including epidermal growth factor (EGF) and transforming growth factor-α. This results in the formation of homo- or heterodimers and activation of receptor tyrosine kinase, which, in turn, leads to signaling cascades and regulation of the expression of the target genes. Studies have shown that interruption of EGF signaling impairs tumor growth (30–34). It has previously been demonstrated that curcumin significantly suppressed gene expression of *EGR1* at transcription and translation levels (35–37). Curcumin successfully reduced the trans-activation activity of the transcription factor EGR-1 by suppressing EGR-1 expression, interrupting the ERK signal pathway and reducing the activity of ELK-1. The authors demonstrated that the reduction of EGR-1 activity plays a critical role in the inhibition of EGFR expression in human colon cancer cells (32). Suppression of EGFR expression and inhibition of receptor tyrosine phosphorylation of EGFR interrupts EGF signaling (38), which collectively contributes to the inhibition of colon cancer cell growth by curcumin. It has been demonstrated that curcumin causes the growth arrest and apoptosis of B-cell lymphoma by downregulation of numerous survival-related genes, including *EGR1*(39). It must be noted, however, that the role of EGR-1 expression in breast cancer is controversial. In a previous study, EGR-1 expression was revealed to be profoundly reduced in human breast cancer tissues and in various cell lines (40,41). In addition, we have to emphasize that since the *in vivo* system is multifactorial and more complicated, directly extrapolating *in vitro* conditions and results into the *in vivo* system may be misleading. Therefore, further studies are required to validate these results.

In conclusion, by applying various bioinformatical approaches we have revealed the decreased expression of pathway elements in the EGF signaling cascade and the decreased expression of the *EGR1* gene on the mRNA level in the MDA-MB 231 human invasive breast carcinoma cell line. To the best of our knowledge we are the first to apply comprehensive GSEA and network analysis to analyze the gene expression profile of curcumin treatment on a breast carcinoma cell line. These observations suggest that curcumin may be an excellent candidate for the prevention and treatment of breast cancer.

## Figures and Tables

**Table I:** Fold changes of gene expression by microarray and qRT-PCR relative to the controls.

Genes	Fold changes obtained by real-time PCR	Fold changes obtained by microarray
*FOS*	2,452,436 (downregulated)	37,162 (downregulated)
*EGR1* (Early growth response 1)	19,027 (downregulated)	10,685 (downregulated)
GDF15 (Growth differentiation factor 15)	42,518 (downregulated)	13,484 (downregulated)

**Table II:** Gene set enrichment analysis.

Pathway set name	NES	NOM P-value	FDR q-value
PDGFPATHWAY	2.1036	0.00	0.0432097
HSA04520_ADHERENS_JUNCTION	1.4830	0.02	0.0433947
EGFPATHWAY	2.0396	0.00	0.0455140
CELL_CYCLE_KEGG	2.0778	0.00	0.0473429
HSA04640_HEMATOPOIETIC_CELL_LINEAGE	−2.1586	0.00	0.0192348
ASBCELLPATHWAY	−1.9279	0.00	0.0338345

A positive normalized enrichment score (NES) indicates correlation with the control group, while negative values indicate correlation with the curcumin-treated group. The nominal (NOM) P-value estimates the statistical significance of the enrichment score for a single gene set. Significant false discovery rate (FDR) and nominal P-values were <5% and 0.05, respectively.
